# Fracture resistance and failure mode of posterior fixed dental prostheses fabricated with two zirconia CAD/CAM systems

**DOI:** 10.4317/jced.52067

**Published:** 2015-04-01

**Authors:** Carlos López-Suárez, Esther Gonzalo, Jesús Peláez, Verónica Rodríguez, María-Jesús Suárez

**Affiliations:** 1 DDS, Researcher, Department of Buccofacial Prostheses, Faculty of Odontology, University Complutense of Madrid, Spain; 2 DDS, PhD, Associate Professor, Department of Buccofacial Prostheses, Faculty of Odontology, University Complutense of Madrid, Spain; 3 MD, DDS, PhD, Professor, Department of Buccofacial Prostheses, Faculty of Odontology, University Complutense of Madrid, Spain

## Abstract

**Background:**

In recent years there has been an improvement of zirconia ceramic materials to replace posterior missing teeth. To date little in vitro studies has been carried out on the fracture resistance of zirconia veneered posterior fixed dental prostheses. This study investigated the fracture resistance and the failure mode of 3-unit zirconia-based posterior fixed dental prostheses fabricated with two CAD/CAM systems.

**Material and Methods:**

Twenty posterior fixed dental prostheses were studied. Samples were randomly divided into two groups (n=10 each) according to the zirconia ceramic analyzed: Lava and Procera. Specimens were loaded until fracture under static load. Data were analyzed using Wilcoxon´s rank sum test and Wilcoxon´s signed-rank test (P<0.05).

**Results:**

Partial fracture of the veneering porcelain occurred in 100% of the samples. Within each group, significant differences were shown between the veneering and the framework fracture resistance (P=0.002). The failure occurred in the connector cervical area in 80% of the cases.

**Conclusions:**

All fracture load values of the zirconia frameworks could be considered clinically acceptable. The connector area is the weak point of the restorations.

** Key words:**Fixed dental prostheses, zirconium-dioxide, zirconia, fracture resistance, failure mode.

## Introduction

The porcelain fused to metal represents the gold standard technique for posterior fixed dental prostheses (FDPs). However, with the increasing demand in esthetic restorations, and the introduction of computer-aided design/computer-aided manufacturing (CAD-CAM) technology in dentistry, in the last years the use of ceramic restorations has been increased (1). Therefore, during the last decade yttrium oxide partially stabilized zirconia (Y-TZP) ceramic is used in dentistry for heavily loaded restoration, showing in vitro and clinical studies promising results (2,3). Zirconia restorations have good fracture resistance but are highly opaque. Therefore, to obtain a natural-looking and improve esthetics the framework should be veneered with porcelain (1), more over, some manufacturers make provision for zirconia colored cores in order to enhance esthetic outcomes (2). It is possible to manufacture the zirconia core in two different ways: milling a fully sintered piece of zirconia, or milling a partially sintered zirconia block and completing the sintering thereafter. Zirconia frameworks sintered after milling has better mechanical properties than densely sintered zirconia (4), but a 20% shrinkage must be allowed to obtain an optimal fit of the frameworks.

Although the good mechanical properties of zirconia allow the construction of posterior FDPs, the core-veneer interface is one of the weakest aspect of these restorations (2,5) so that, delamination or chipping of the veneering porcelain has been described as the most frequent reason for the failure of zirconia FDPs (3,6). It is important to understand how the veneer and framework material interact in a multi-layer configuration, so the study of veneered structures can provide information about the fracture resistance of each component and the failure mode and origin (1).

The aim of the present study was to compare the fracture resistance (FR) of two zirconia CAD/CAM systems with their corresponding porcelains and the failure mode of 3-unit zirconia posterior fixed dental prostheses (FDPs) with an intermediate pontic. The null hypothesis was, that no differences would be found in the fracture resistance and the failure mode between the zirconia systems.

## Material and Methods

Twenty standardized specimens with 2 abutments and screwed onto a platform (30 mm in length, 17 mm in width, and 4.5 mm in thickness) were prepared from stainless steel to receive posterior 3-unit FDPs with an intermediate pontic (spanning the first premolar to the first molar). The abutments were prepared with 5 mm in height, a 1 mm wide chamfer, and a 6 degrees angle of convergence of the axial walls. The specimens were used as working dies and randomly divided in 2 groups (n=10 each). Two zirconia materials and two veneering porcelain were used: Group 1 (L) Lava All-ceramic System and Lava Ceram (respectively) (3M ESPE, Seefeld, Germany) and Group 2 (P): Nobel Procera Zirconia and NobelRondo (respectively) (Nobel Biocare, Zurich, Switzerland). The size of the connector area was 3 mm x 3 mm and the frameworks thickness was 0.5 mm, with a space of 50 µm for the cement agent. The digitization of the specimens was performed using an optical scanner in the L group (Lava Scan, 3M ESPE, Seefeld, Germany) and a mechanical scanner in the P group (Procera Forte, Nobel Biocare, Zurich, Switzerland).

Samples fabricated with each zirconia system were luted onto the master dies at room temperature with glass ionomer cement (Ketac Cem Easymix, 3M ESPE, Seefeld, Germany). The cement was placed on the axial surfaces of the abutments and a standardized load of 10 N was applied for 10 minutes with a dynamometric key (USAG 820/70, SWK Utensilerie, Milano, Italy) to ensure the correct distribution of the cement and to seat the FDPs properly. The cemented FDPs were stored in water for 1 week at 37 °C.

All FDPs were subjected, according to the ISO 6872:2008, with a three-point bending test until fracture using a universal testing machine (ME 405/10, SERVOSIS SA, Pinto, Spain) at a crosshead speed of 0.5 mm/min. Axial compressive loads were applied at the central fossa of the FDPs´ pontic. Data of the veneering ceramic fracture (partial fracture) and total fracture of the FDPs were automatically recorded. The force was measured in Newton (N). After testing the location of the fracture was examined visually and with a stereomicroscope (x15).

The Wilcoxon rank sum test and the Wilcoxon signed-rank test were run for FR (N) comparisons. Statistical significance was set at *P* < 0.05. All statistical analyses were handle with SAS 9.2 (SAS Institute Inc, Cary, NC, USA).

## Results

 Table 1 shows the mean and standard deviation values of fracture resistance for the experimental groups. The L and the P groups recorded comparable total FR with no significant differences between them (*P*=0.07). Significant differences in the fracture resistance of the veneering ceramic were recorded between both groups (*P*=0.0023). The fracture resistance of the veneering ceramic and the total FR were significantly different within each experimental group (*P*=0.002). The L group showed the highest mean value.

**Table 1:**
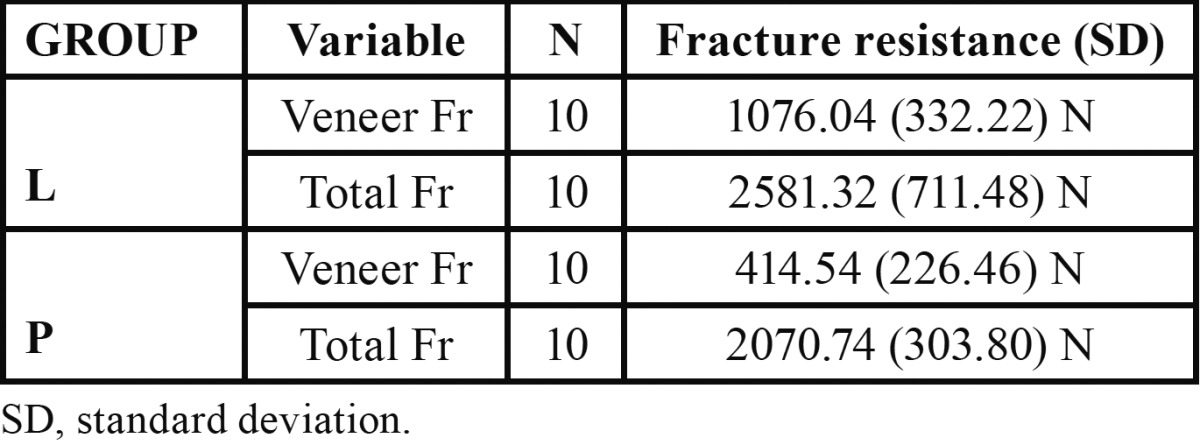
Fracture resistance (in N) of veneer ceramic and total fracture of Lava and Procera group.

**Figure 1:**
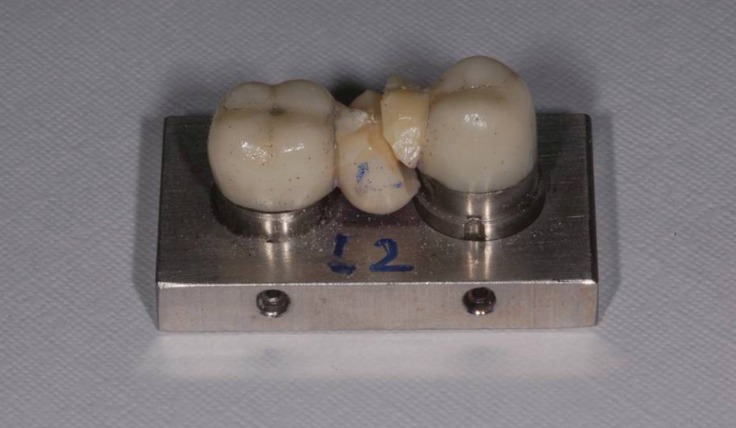
Representative fracture pattern of a specimen from the Lava group.

**Figure 2:**
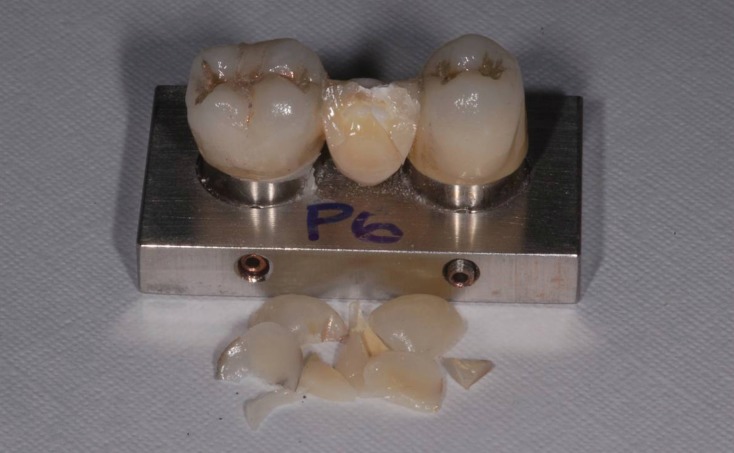
Partial fracture of the porcelain layer of a specimen from the Procera group.

Regardless of the zirconia system used, the failure mainly occurred at the cervical area of the connector (80%) (Fig. 1). In both groups, occurred partial fracture in 100% of the samples, in which the porcelain veneer failed before fracture of the framework material, resulting in delamination of the porcelain layer. Seven specimens of the L group exhibited cohesive fracture within the veneering porcelain, and 3 specimens exhibited adhesive failure. In the P group, 6 specimens failed adhesively and 4 specimens exhibited mixed failure (Fig. 2).

## Discussion

The present study analyses the fracture resistance of two zirconia systems and no differences were observed in total fracture, but differences were shown for veneering ceramic between the groups thus the data support the partially acceptance of the null hypothesis.

Little in vitro studies have been carried out regarding the strength of frameworks and porcelain veneering on zirconia posterior FDPs. The study results indicate that in all the specimens and in both zirconia systems the veneer porcelain fractures at a lower load than the framework, with significant differences in fracture values between the veneer and the framework. These results are in accordance with previous studies in which failure of the porcelain layer before failure of the framework material was observed (1,7-11). The porcelain delamination observed is probably related to zirconia superior mechanical properties (1). The results are also in accordance with those of clinical studies in which the main problem identified in posterior zirconia FDPs, is the chipping of the veneer porcelain (3,12-15).

In the present study, the Procera group revealed predominantly adhesive failure between the zirconia core and the veneer ceramic in 6 specimens and adhesive failure mode in 4 specimens, but in the Lava group, the failure pattern was predominantly cohesive, in agreement with previous studies in which the most frequent type of fracture occurs within the porcelain veneer rather than at the porcelain-zirconia interface (8,9,11,16-20). However, as was reported in a previous study the stereomicroscope evaluation may not be sufficient to truly determine the mode of fracture (9).

Different factors may influence and/or cause the fracture of the veneering porcelain as differences in termal expansión coefficients between core and ceramic, flexural strength of the veneering ceramic, firing shrinkage of ceramic, porcelain thickness, surface treatament of the framework, flaws on veneering and pour wetting by veneering on core (2,3,9,12,15). Special ceramics are nowadays developed for zirconia in order to minimize this unfavorable aspect (2).

Despite the many disadvantages of in vitro studies, it is important to evaluate isolated mechanical properties under standardized conditions (21). In this in vitro study, the methods used were chosen to reflect the clinical situation as far as possible. The tooth preparation design and dimensions of both zirconia groups are identical, and the veneer porcelain was fired according to the manufacturer´s, recommendations, with appropriate dimensions and an identical layered build-up technique, thus it is possible to make comparisons between the two zirconia systems. One important factor to take into consideration for the fracture resistance of the restorations is the anatomy of the framework. It is important to avoid the occurrence of areas with too little or too great a veneer thickness, which might reduce the restorations´ resistance (1,20,22-24), thus in the present study the design of the framework was anatomically shaped. The present study was done under compressive testing and although does not reproduce conditions in the oral environment as cyclic studies, the results of this type of test provide valid information (11,20), and previous studies showed that cyclic loading conditions does not affect the resistance of the material (9,22).

The most common fracture pattern of tested zirconia-based FDPs was at the loading point and through one or both connectors, being the iniciation of fracture in the gingival embrasure (15,22,25-27). This was in accordance with the present results in which the fracture was initiated from the gingival surface of the connector and propagated toward the pontic. Thus the results support that the connector design appears to be crucial for the fracture resistance and longevity of zirconia FDPs and should be taken into account when designing zirconia-based FDPs as previously reported (6,15).

Further laboratory and clinical studies are needed to evaluate the veneering porcelain on the fracture process of zirconia FDPs.

## Conclusions

Within the limitations of this in vitro study, it can be concluded that the fracture resistance of the veneering ceramic of zirconia structures depends on the zirconia system used. The Lava system exhibited the greatest resistance of the veneering ceramic. However, the zirconia system had no effect on the ultimate resistance of zirconia frameworks. All tested groups demonstrated clinically acceptable fracture load values.
